# PhoR/PhoP two component regulatory system affects biocontrol capability of *Bacillus subtilis* NCD-2

**DOI:** 10.1590/S1415-47572010005000032

**Published:** 2010-06-01

**Authors:** Qinggang Guo, Shezeng Li, Xiuyun Lu, Baoqing Li, Ping Ma

**Affiliations:** Institute of Plant Protection, Hebei Academy of Agricultural and Forestry Sciences, Integrated Pest Management Centre of Hebei Provence, BaodingChina

**Keywords:** *Bacillus subtilis*, antagonism, *Verticillium dahliae*, mini-Tn*10*

## Abstract

The *Bacillus subtilis* strain NCD-2 is an important biocontrol agent against cotton verticillium wilt and cotton sore shin in the field, which are caused by *Verticillium dahliae* Kleb and *Rhizoctonia solani* Kuhn, respectively. A mutant of strain NCD-2, designated M216, with decreased antagonism to V. dahliae and *R. solani*, was selected by mini-Tn*10* mutagenesis and *in vitro* virulence screening. The inserted gene in the mutant was cloned and identified as the *phoR* gene, which encodes a sensor kinase in the PhoP/PhoR two-component system. Compared to the wild-type strain, the APase activities of the mutant was decreased significantly when cultured in low phosphate medium, but no obvious difference was observed when cultured in high phosphate medium. The mutant also grew more slowly on organic phosphate agar and lost its phosphatidylcholine-solubilizing ability. The suppression of cotton seedling damping-off *in vivo* and colonization of the rhizosphere of cotton also decreased in the mutant strain when compared with the wild type strain. All of these characteristics could be partially restored by complementation of the phoR gene in the M216 mutant.

## Introduction

Cotton is one of the major crops in China as well as in the world. Verticillium wilt, caused by *Verticillium dahliae* Kleb is a devastating disease in cotton, causing heavy economic losses globally (Tjamos *et al.*, 2000). This disease is difficult to control due to the absence of resistant varieties, the long viability of the resting structures, and the inability of fungicides to inhibit the pathogen once inside the xylem (Fradin and Thomma, 2006). Therefore, research on alternative management strategies for cotton verticillium wilt control is important. One alternative is the use of antagonistic microorganisms which can suppress soil-borne pathogens in the rhizosphere of cotton (Weller *et al.*, 2002; Tjamos *et al.*, 2004). There are several reports on treatments with such beneficial organisms providing a pronounced reduction in plant disease, both in greenhouses and in the field. One example is the gram-positive bacterium *Bacillus subtilis*, a bacteria widely distributed in nature, and which has been found to possess bio-control potential for a variety of phytopathogenic fungi (Leifert *et al.*, 1995; Podile and Prakash, 1996). The mechanisms by which *B. subtilis* reduces plant diseases include antagonism of fungal pathogens by competing for niche and nutriments (Handelsman and Stabb, 1996), by producing fungitoxic compounds (Toure *et al.*, 2004; Leclere *et al.*, 2005), and stimulating the defensive capacities of the host plant (Ongena *et al.*, 2004).

Two-component regulatory systems are major mechanisms by which *B. subtilis* strains sense environmental stimuli and respond by specific gene activation (Parkinson and Kofoid, 1992). These systems depend upon histidine kinases that serve both to sense a particular stimulus and to transmit the information to the response regulator via a phosphoryl-transfer reaction, and to up- or down-regulate the expression of specific target genes (Hoch, 2000). To date, more than 35 two-component regulatory systems have been identified in *B. subtilis* by genome sequencing, these functioning in the adaptation to environmental stress (Darmon *et al.*, 2002), the production of secondary metabolites (Martin, 2004), and cell division (Fukuchi *et al.*, 2000). Further functions may be discovered by mutagenesis studies.

PhoP/PhoR is one such important two-component regulatory system in *B. subtilis* and other gram-negative and gram-positive bacteria. In response to phosphate deficiency, PhoR, a histidine sensor kinase, phosphorylates its cognate response regulator PhoP (PhoP~P) and either induces or represses Pho regulon genes by binding of PhoP~P to Pho box sequences: direct repeats of TT(A/T/C)ACA with a 5 ± 2-bp spacer (Eder *et al.*, 1999). At present, more than 30 genes classified as Pho regulon are controlled by the PhoP/PhoR system have been identified by proteomics and transcriptional studies (Antelmann *et al.*, 2000; Pragai *et al.*, 2004).

The *B. subtilis* NCD-2 was selected from rhizosphere of cotton and is an antagonist of *V.dahliae* and *Rhizoctonia solani*, the phytopathogens which cause damping-off disease in cotton seedlings. This strain was selected for further research because of its high efficacy in controlling cotton verticillum wilt and cotton seedling damping-off in the field, although its antifungal activity is not as effective as some other biocontrol strains (Li *et al.*, 2005). Previous research also confirmed that the NCD-2 strain has the ability to solubilize phosphatidylcholine (Ma, unpublished). In this study, we used transposon mutagenesis to clone functional genes involved in the biocontrol activity of the NCD-2 strain. We report on the identification and characterization of a gene that is part of the PhoP/PhoR two-component system in regulating the antifungal ability of NCD-2 against *V. dahliae* and phosphatidylcholine-solubilizing ability, as well as suppression of cotton damping-off.

## Materials and Methods

###  Bacteria strains, growth conditions, and plasmids

The bacterial strains and plasmids used in this study are listed in Table 1. *Escherichia coli* DH5α was used as the host for plasmid construction. All strains were grown in Luria Bertani (LB) medium supplemented when necessary with the appropriate antibiotic (for *E. coli*, ampicillin at 100 μg/mL, erythromycin at 100 μg/mL, tetracycline at 20 μg/mL; for *B. subtilis*, chloramphenicol at 5 μg/mL, erythromycin at 1 μg/mL, tetracycline at 20 μg/mL).

###  Mini-Tn*10* mutagenesis and generation of the transposon library

The *B. subtilis* strain NCD-2 was transformed with the plasmid pHV1249 by the protoplast fusion method (Martin *et al.*, 1981), whereupon transformants were selected on LB agar containing 5 μg/mL chloramphenicol (Cm) and 1 μg/mL erythromycin (Em). Individual colonies of NCD-2 transformants carrying intact plasmid pHV1249 were inoculated into LB culture containing 5 μg/mL Cm. The cultures were grown with shaking at 30 °C. When the culture reached the logarithmic phase (optical density of 0.6-0.8 at 660 nm), it was diluted 100-fold in water, and 100 μL samples of the diluted culture were spread onto LB agar plates containing 5 μg/mL Cm, and then incubated at 51 °C for the selection of transposants (Tsuge *et al.*, 1999). Thermoresistant Cm^r^ colonies were regarded as mini-Tn*10* insertional mutants and the Cm^r^Em^s^ colonies were selected for the antagonism assay.

###  Screening of mutants for loss of antifungal ability against *Verticillium dahliae*

For *in vitro* screening of transposants for loss of antagonistic activity against *V. dahliae*, well diffusion inhibition assays were conducted as described by Cintas *et al.* (1995) with the following modifications. The *V. dahliae* was grown for 4- to 6 days in potato dextrose broth (PDB) at 25 °C and then filtrated by two layers of gauze. Soft agar was made by adding 0.7% agar to PDB, and to create an overlay. *V. dahliae* was added to the soft agar in a ratio of 2 mL of fungal culture per 100 mL soft agar (10^6^ CFU mL^- 1^). From this mixture, 8 mL was overlaid onto each base plate (PDB containing 1.5% agar) and allowed to solidify completely. Wild type *B. subtilis* strain NCD-2 and transposants were transferred with toothpicks onto the layered medium, one wild-type strain and eight transposants per dish. All plates were incubated for 3 to 4 days at 30 °C. The antifungal ability of both transposants and wild-type strain against *V. dahliae* was recorded as the size of the zone of fungal inhibition. Mutants with decreased antagonism against *V. dahliae* were also used to test the antagonism against *R. solani* by the dual-culture method.

###  Southern blot hybridization

For Southern blot hybridization, an internal 700-bp fragment of mini-Tn*10* was obtained by PCR with the primers mini-Tn10S (5'CGTTATTTGAGTTTATCACCCT3') and mini-Tn10A (5'TAAGTCTTCCCTTGTTATTGTG3'). This fragment was purified and labeled using a DIG High Prime DNA Labeling and Detection Starter Kit (Roche, Indianapolis). *B. subtilis* strain DNA was cleaved with *Eco*RI and *Pst*I (TaKaRa), whereupon the fragments were separated on a 0.8% agarose gel, transferred to a nylon membrane, and then cross-linked by UV irradiation. Hybridization using the labeled fragment as a probe was carried out according to the instructions of the DIG-DNA Labeling and Detection Kit.

###  Cloning and sequencing of mini-Tn*10* insertions and complementation study

The flanking sequences of the mini-Tn*10* insertion were obtained by a nested PCR method. Chromosomal DNA from the mutant was extracted and used as a template with the anchor primer AP1 (supplied by TaKaRa company) and the specific primers SP1 (5'CATTGCTCTGAAAGCGGGAACG3'), SP2 (5'GCATCGTATTGCCCGAACAGATAA3'), and SP3 (5'GGCCAAGTTCGGTAAGAGTGAGA3'), which had been designed according to the mini-Tn*10* sequence. The final products amplified with AP1 and SP3 primers were purified and sequenced, the homologues were identified by blastx programs.

**Figure 1:**
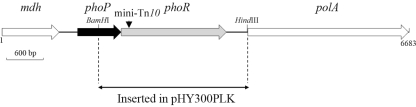
Schematic diagram of *B. subtilis* NCD-2 *mdh*, *phoP*, *phoR* and *polA* genes. Single-headed arrows represent the location and orientation of genes in the *B. subtilis* NCD-2 chromosome. Main restriction enzyme sites are shown, the mini-Tn*10* in M216 mutants being inserted into the *phoR* gene.  For M216 mutant phoR gene complementation, the gene itself was inserted into a pHY300PLK plasmid.

To complement the M216mutant, a 2.64-kb DNA fragment containing the *phoR* gene was amplified with the primer set phoRS (5'CCaagcttTTTTCTGTCTGCCGCCTTTA3') and phoRA (5'CGggatccACCAAGCCGTTCAGTCCAAG3') (underlined letters indicate *Hind*III and *Bam*HI restriction sites, respectively). The resulting PCR product was digested with *Hind*III and *Bam*HI, and then cloned into the pHY300PLK plasmid to yield pHY300PLK-R (Figure 1). The recombinant plasmid pHY300PLK-R was first transformed into *E. coli* DH5α , extracted and then transferred to the M216 mutant by electrotransformation for the complementation study.

###  Measurements of alkaline phosphatase (APase) activity and phosphatidylcholine-solubilizing ability

In order to measure APase activity, cells were grown in low-phosphate (0.065 mM phosphate) or high-phosphate (10 mM phosphate) medium for 1, 2 or 3 days. Cells were harvested by centrifugation at 12,000 rpm for 15 min, washed with sterile distilled water, suspended with 50 mM Tris (pH 7.5) and dispersed ultrasonically (20 kz, 2 A, 60 Hz) for 40 min in ice water (Raso *et al.*, 2008), before measuring APase activity by using Olsen's method (Olsen and Sommers, 1982). Qualitative comparison of phosphatidylcholine-solubilizing ability were performed on Mongina organic phosphate agar plates. *B. subtilis* strains were transfered to Mongina organic phosphate agar plates (0.5 g/L (NH_4_)_2_SO_4_; 0.3 g/L NaCl; 0.3 g/L MgSO_4_.7H_2_O; 0.3 g/L KCl; 0.03 g/L FeSO_4_.7H_2_O; 0.03 g/L MnSO_4_.4H_2_O; 5 g/L CaCO_3_; 10 g/L glucose; 1.5% agar; 85 mL/L egg yolk solution), and to assess growth and qualitatively compare phosphatidylcholine-solubilizing abilities after incubation at 37 °C for 5 to 7 days.

###  Root colonization and biocontrol of cotton seedling damping-off

To evaluate the biocontrol capability against cotton seedling damping-off and root colonization, the *B. subtilis* strains NCD-2/pHY300PLK and M216/pHY300PLK were used to represent the *B. subtilis* NCD-2 wild-type and M216 mutant, respectively. All strains were grown at 30 °C for 30 h in LB medium, cell were harvested by centrifugation at 12,0000 rpm for 30 min, and the cells were adjusted with sterile distilled water to obtain the desired bacterial concentration for seed treatment (OD_550_ = 5). Cotton seeds (*Gossypium hirsutum* cv. Jimian99B) were surface sterilized and germinated before sowing by washing three times with sterile distilled water and soaking for 15 min in the bacterial suspension mentioned above or in sterile distilled water as a control. In every experiment, 84 seeds were used for each treatment. The seeds were dried under the laminar flow and sown in plastic packs containing growth substrate (sterilized soil: vermiculite 3:1), previously infected with *R. solani* by mixing with a suspension of mycelial fragments (10^5^ propagules.g^-1^ soil). The trays were incubated in a greenhouse to maintain the temperature at 28 °C at 95% relative humidity with a photoperiod of 16 h.

The initial bacterial inoculum per seed was determined by suspending 10 seeds in 5 mL sterile water, vortexing for 10 min and then spreading 50 μL serially diluted samples on an LB agar medium containing 20 μg/mL tetracycline. The plates were incubated at 30 °C for 24 h, and the representative colonies were subsequently counted.

To monitor *B. subtilis* NCD-2 and its derivative strains colonization in the cotton rhizospheres, at 8 and 16 days after sowing, three plants per treatment were carefully removed from the soil and roots with adhering soil were cut and tested by plating-PCR with *B. subtilis* NCD-2 strain-specific primers RT-phoRA (5'ACTTGGCTTCGCTTCTTGAT3'), and RT-phoRS (5'TTTGGGAATCAGCCGCTCTC3'), (the result of a specific unpublished assay). Briefly, large aggregates of soil were gently removed from the roots and rhizosphere soil were collected and air-dried. The roots were carefully washed in 2 mL of 0.9% NaCl solution to collect the still adhering rhizosphere soil. The roots were then removed from the solution and the bacterial cells were extracted for 5 min using a rotary shaker at maximum speed. The bacteria containing solutions were then serially diluted (1: 10-1: 10^3^) and 100 mL of each dilution were plated on LB agar plates supplemented with 20 μg/mL tetracycline (Sigma-Aldrich). After 24 h of incubation at 50 °C, colony numbers were recorded, and colonies similar to the wild-type strain were directly dissolved in PCR reaction tubes and tested with the RT-phoRA/RT-phoRS primer pair. After sampling, the remaining cotton seedlings were used for evaluating the biocontrol capability of *B. subtilis* NCD-2 and its derivative strains.

###  Statistical analysis

Three replicates were carried out to assess *in vivo* root colonization and bio-control capability of both *B. subtilis* NCD-2 and its derivative strains*.* All data were analyzed by two-way ANOVA using SAS for Windows ver. 9.0 software. Differences were considered to be significant at a 95% (or higher) confidence level.

## Results

###  Isolation and identification of mutants defective in antifungal ability against *V. dahliae* and *R. solani*

The NCD-2 strain, known to be well-capable of suppressing cotton verticillum wilt and cotton damping-off in seedlings, was identified as a *Bacillus subtilis* strain by 16S rDNA sequencing (GenBank accession number FJ624484) and biochemical assaying. In order to clone the functional gene associated with antifungal activity from the NCD-2 strain, a transposon mutant library of *B. subtilis* NCD-2 was constructed with mini-Tn*10,* whereby more than 4000 Cm^r^Em^s^ colonies were selected and screened for antibiosis activities against *V. dahliae* mycelial growth. Four mutants with decreased antagonism against *V. dahliae* were purified. As described in materials and methods, *in vitro* testing revealed that these mutants also expressed decreased antagonism against *R. solani*. Southern hybridization with a mini-Tn10 DNA fragment as a probe showed that all these mutants contained a single copy of inserted transposon which possibly localized at the same chromosomal location because the size of hybridized fragments in each mutant was identical (data not shown). One such mutant was designated as M216 and employed for further study.

###  Cloning and sequencing of the genes involved in antifungal activity of NCD-2

**Figure 2:**
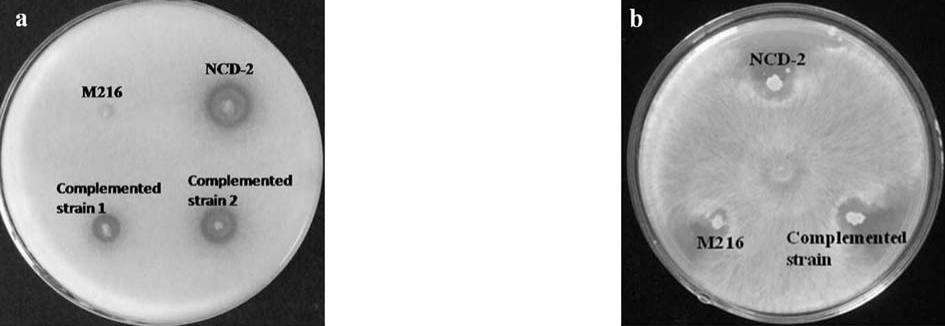
Inhibition by *B. subtilis* NCD-2 wild-type and its derivative strains of in vitro growth of *Verticillium dahliae* (a) and *Rhizoctomia solani* (b).  Double-layer culture assaying was employed to check *V. dahliae* inhibition, with antifungal capability being recorded according to the zone surrounding the colony. Dual-culture assaying was carried out to check *R. solani* inhibition, whereas antifungal capability was recorded according to the inhibition zone surrounding the colony.

A 1.5 kb DNA fragment flanking the mini-Tn*10* in the M216 mutant was obtained by nested-PCR. Sequencing analysis showed significant similarity (98%) between the transposon-disrupted gene in the M216 mutant and the *phoR* gene in *B. subtilis* 168, also known as a sensor kinase in the PhoP/PhoR two-component regulatory system (Figure 1). By using a complemented strain containing a plasmid-borne *phoR* gene, we showed that the antagonistic activity of M216 mutant against *V. dahliae* and *R. solani* could be significantly restored (Figure 2).

In *B. subtilis*, the PhoR/ PhoP two-component system regulates the biosynthesis of certain secondary metabolites at the transcriptional and post-transcriptional levels when undergoing phosphate deprivation. The sequence of PhoP/PhoR two-component system was obtained in strain NCD-2 through sequencing of its chromosomal DNA. The *phoR* gene (GenBank accession number EU165270) encodes a protein of 579 amino acids which has 98% identity with *phoR* of *B. subtilis* 168. The *phoP* gene of NCD-2 (GenBank accession number EU188794) encodes a protein of 239 amino acids which has 98% identity with *phoP* of *B. subtilis* 168.

###  Quantitative measurements of APase activity

**Figure 3:**
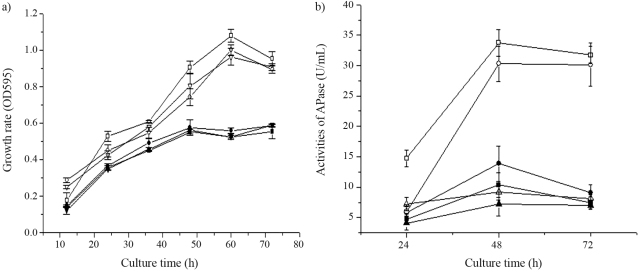
Growth and alkaline phosphatase (APase) activities of *B. subtilis* NCD-2 and its derivative strains during growth in both a high and low phosphate medium.  Samples were removed periodically, and APase activities assayed in cell extracts. The values are averages of three replications, with mean values ± SD indicated. Squares, NCD-2 wild-type; triangles, M216 mutant; circles, M216 mutant complemented strain. Open symbols, cell growth in a high phosphate medium; closed symbols, cell growth in a low phosphate medium.

*B. subtilis* can produce vegetative APase when phosphate becomes growth limiting (Bookstein *et al.* 1990) in a pathway that is mediated by the PhoP/PhoR two-component regulatory system (Hulett *et al.*, 1990). Since the APase of *B. subtilis* NCD-2 is mainly located intra-cellularly and only very small amounts can be detected extra-cellularly (data not shown), we only compared the activities of intracellular APase in *B. subtilis* NCD-2 wild-type, mutant and complemented strains cultured in high and low phosphate medium. The APase activity of *B. subtilis* NCD-2 wild-type was significantly lower in high phosphate medium than in low phosphate medium, consistent with the results of previous research (Bookstein *et al.*, 1990). When grown in high phosphate medium, no significant differences in APase activities among NCD-2 wild-type, mutant and complemented strains were observed. In low phosphate medium, however, the APase activity of M216 mutant was much lower than that of NCD-2 wild-type, and the APase activity of M216 mutant was nearly completely restored when complemented with the *phoR* gene (Figure 3a). The growth of NCD-2 wild-type and derivative strains were also compared, the results indicating no significant difference when cultured both in high and low phosphate media (Figure 3b).Thus, the result indicated that the *phoR* gene is involved in the APase activities in the NCD-2 strain.

###  Qualitative measurements of phosphatidylcholine-solubilizing ability

**Figure 4:**
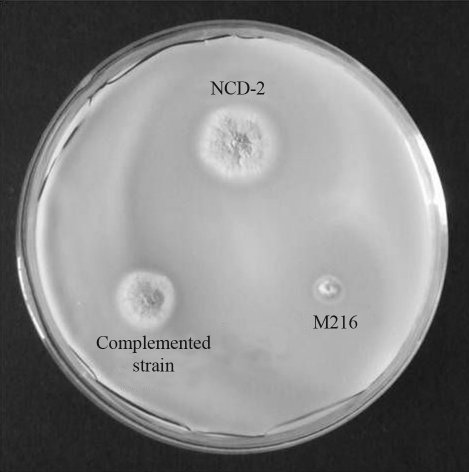
Growth and phosphatidylcholine-solubilizing ability of *B. subtilis* NCD-2 wild-type strain and derivative strains.  The growth and phosphatidylcholine-solubilizing ability were recorded according to the diameter of the colony and zone around the colony, respectively, the results being recorded after incubation for 5 days at 37 °C.

In *B. subtilis*, *phoD* encodes a phosphodiesterase that may be involved in the hydrolysis of phosphatidylcholine, whereas PhoP/PhoR positively regulates *phoD* expression (Hulett *et al.*, 1990). Therefore, in this study we asked if the PhoR/PhoP two-component regulatory systemis involved in the abilities of the *B. subtilis* NCD-2 strain to solubilize and utilize phosphatidylcholine, as demonstrated by the clear zone surrounding each colony and the growth on organic phosphate culture media. Compared with the NCD-2 wild-type strain, the M216 mutant had decreased ability to solubilize phosphatidylcholine , and lost the ability to utilize phosphatidylcholine. Both abilities in the M216 mutant were restored when complemented with *phoR* (Figure 4), indicating that they are specifically regulated by this gene in the NCD-2 strain. However, whether the regulation of *phoR* is mediated by *phoD* should be investigated in future studies.

###  Biocontrol of cotton seedling damping-off

The NCD-2 strain possesses antagonistic properties against *R. solani* and can effectively suppress cotton seedling damping-off in the field. Pre-treatment of cotton seeds with vegetative cells of the wild-type NCD-2 strain provided consistent protection against cotton damping-off disease, as observed through three independent experiments. By assessing the percentage of healthy seedlings, the capacity in suppressing cotton seedling damping-off was less in M216 mutants than in the wild-type NCD-2 strain, although becoming partially restored in the complemented M216 mutant (Table 2).

###  Monitoring *B. subtilis* NCD-2 and derivative strains on cotton roots

After application on cotton seeds at a concentration of 1.08 x 10^5^-1.39 x 10^5^ CFU/seed, the population level of *B. subtilis* NCD-2 and derivative strains in cotton rhizospheres was evaluated through selection in antibiotics and by plating-PCR. More than 96% of the colonies tested by PCR in the plating method were identified as *B. subtilis* NCD-2 or derivative strains. Eight days post-sowing, there was no significant difference in the population density of *B. subtilis* NCD-2 wild-type and derivative strains in the rhizospheres , but 16 days after sowing the population density of the M216 mutant was lower only at 114 CFU (g wt soil)^-1^, whereas the population density of the NCD-2 wild-type strain was 3.08 x 10^3^ CFU (g wt soil)^-1^, thus significantly higher than the the population density of M216 mutant. The population density of the *phoR* complemented strain was also higher than that of M216 mutant, but not significantly different when compared to *B. subtilis* NCD-2 wild-type (Table 3). Therefore, it can be inferred that colonization of *B. subtilis* NCD-2 was regulated by the *phoR* gene.

## Discussion

The PhoP/PhoR two-component system regulates the biosynthesis of some secondary metabolites at the transcriptional and post-transcriptional level under phosphate-deprived conditions (Martin, 2004). In *Streptomyces lividans*, several antibiotics have been shown to be negatively regulated by the PhoR/PhoP system (Sola-Landa *et al.*, 2003). In *B. subtilis*, more than 30 genes were classified as belonging to the pho regulon and being controlled by the PhoR/PhoP system (Martin, 2004), and by genome-wide transcriptional analysis increasingly more genes were identified as belonging to the pho regulon (Allenby *et al.*, 2005), However, to our knowledge, no antibiotics have been identified as being controlled by this two-component system in *B. subtilis*. In this study, the PhoP/PhoR two-component regulatory system was cloned from *B. subtilis* NCD-2 and the *phoR* gene was disrupted by mini-Tn*10*. We found that the selected M216 mutant strain had decreased antagonism against *V. dahliae* and *R. solani*, and complementation of *phoR* could restore the antagonistic activity of this mutant. Therefore, we concluded that the antagonistic activities of *B. subtilis* NCD-2 against *V. dhaliae* and *R. solani* are controlled by the PhoR/PhoP two-component system. However, we do not yet know what antibiotics or metabolites are controlled by the PhoR/PhoP system.

*B. subtilis* can produce APase or phosphodiesterase (PDase) when phosphate becomes growth-limiting (Bookstein *et al.*, 1990), this process being mediated by the PhoR/PhoP two-component system (Hulett *et al.*, 1990). We compared the APase activities from the intracellular compartment where it is mainly located in *B. subtilis* NCD-2 wild-type, mutant and complemented strains cultured in high and low phosphate media. APase activities in the NCD-2 wild-type strain were higher when encountering phosphate starvation, and lower in the *phoR* disrupted strain than in the NCD-2 wild type when cultured in low phosphate medium but not different when cultured in high phosphate medium, thus confirming *phoR* involvement in the regulation of APase genes in the NCD-2 strain.

In *B. subtilis*, encoding APase genes (*phoA*, *phoB*) and the PDase gene (*phoD*) are induced by PhoR/PhoP regulators on undergoing phosphate starvation (Le Hegarat and Anagnostopoulos, 1973). *phoD* encodes a phosphodiesterase possibly involved in the hydrolysis of phosphatidylcholine, whereas *phoA*, and *phoB* encode alkaline phosphatases (APases) which facilitate the recovery of inorganic phosphate from organic sources (Hulett *et al.*, 1990). As *B.subtilis* NCD-2 has the ability of solubilizing phosphatidylcholine and the *phoR* disrupted mutant lost phosphatidylcholine-solubilizing ability, solubilizing phosphatidylcholine in NCD-2 may due to PDase. The *phoR* disrupted mutant grew slower on Mongina organic phosphate culture medium than NCD-2 wild type, a result of less phosphate obtained by mutant. This result indicated that the PhoP/PhoR two-component system may be involved in phosphatidylcholine-solubilization.

Rhizosphere competence and colonization are important prerequisites in effective biological control by bio-control agents (Compant *et al.*, 2005). Bio-control agent competence greatly depends on the ability to either take advantage of a specific environment or on their abilities to adapt to changing conditions (Ravel and Cornelis, 2003). Soluble phosphorus in the soil is usually very scarce, although other organic phosphate compounds in the form of phosphodiesters may also exist in soil (Rodriguez and Fraga, 1999). Thus we hypothesize that NCD-2 is capable of growing rapidly and becoming predominant by hydrolyzing phosphodiesters and acquired inorganic phosphate from the soil. From a previous study, it was deduced that *B. subtilis* NCD-2 has the capacity of efficiently colonizing the surface of cotton roots (Ma, unpublished). In this study, the cell density of *B. subtilis* NCD-2 and derivative strains in the rhizosphere of cotton were compared, whereby it was found that the population of *phoR* disrupted mutants therein decreased sixteen days after sowing, although this tendency could be reversed by complementation with *phoR*. Therefore, rhizosphere competence and colonization may be other factors controlled by the PhoR/PhoP system, besides contributing to the effective suppression of cotton verticillum wilt and cotton seedlings damping-off by NCD-2 in the field.

## Figures and Tables

**Table 1:** Bacterial strains or plasmids used in this study.

Strain or plasmid	Genotype or characteristics	Source
*B. subtilis*		
NCD-2	Wide-type	Isolated from rhizophere of cotton
M216	*phoR*::mini-Tn*10* in strain NCD-2, Cm^r^	This study
Fungal		
*V. dahliae*	Cause cotton Verticillium wilt	Lab stock
*R. solani* Kuhn	Cause cotton seedling damping-off	Lab stock
Plasmids		
pHV1249	Delivery plasmid for mini-Tn*10* transposon, Cm^r^ Em^r^ Ap^r^	Lab stock
pHY300PLK	*E.coli* and *Bacillus* Shuttle vector, Ap^r^ Tet^r^	TaKaRa
pBluescript II SK ±	Cloning vertor, Ap^r^	Stratagene

Tet^r^, tetracycline resistance; Cm^r^, chloramphenicol resistance; Ap^r^, ampicillin resistance; Kan^r^, kanamycin resistance.

**Table 2:** Effect of strain NCD-2 and its derivative strain on the reduction of damping-off of cotton plants caused by *Rhizoctonia solani* Kuhn in greenhouse.

Treatments	Healthy seedlings(%)^*^
Control	53.57 ± 8.2a
M216	58.60 ± 6.7 ab
Complemented strain	70.01 ± 8.0b
NCD-2 wild type	71.82 ± 7.1b

The percentage of healthy seedlings was recorded 2 weeks after inoculation, with sterilized-water-treated seeds as control. The values are averages of three replications, the mean values ± SD are indicated. In the same column, the same letters are not significantly different (p ≤ 0.05) on using ANOVA least-significant-difference testing.

**Table 3:** Colonization of *B. subtilis* NCD-2 and derivative strains on root of cotton seedlings.

Treatments	Initial bacterial density (CFU per seed)*	Bacterial cell count (CFU g-1 soil)*	
		8 days	16 days
NCD-2/pHY300PLK	1.19 x 10^5^ a	3.98 x 10^3^ a	3.08 x 10^3^ a
Complemented strain	1.08 x 10^5^ a	3.87 x 10^3^ a	2.29 x 10^3^ ab
M216/pHY300PLK	1.39 x 10^5^ a	2.95 x 10^3^ a	0.14 x 10^3^ b

*The values are averages of three replications, with mean values indicated. In the same column, the same letters are not significantly different (p ≤ 0.05), on using ANOVA least-significant-difference testing.
